# Phenotypic detection of Extended Spectrum β-Lactamases (ESBL) among gram negative uropathogens reveals highly susceptibility to imipenem

**DOI:** 10.12669/pjms.35.4.207

**Published:** 2019

**Authors:** Isra Mohammed, Elfadil Abass

**Affiliations:** 1 Isra Mohammed, College of Graduate Studies, Sudan University of Science and Technology, P.O. Box 407, Khartoum, Sudan; 2 Elfadil Abass, Department of Clinical Laboratory Science, College of Applied Medical Sciences, Imam Abdulrahman Bin Faisal University, P.O. Box 2435, Dammam, Saudi Arabia

**Keywords:** ESBL, Gram Negative, Phenotypic Detection, Pregnant women

## Abstract

**Objective:**

We aimed to determine antibiotic susceptibility patterns of ESBL- and non-ESBL bacteria isolated from pregnant women with UTI in antenatal wards in Khartoum State, Sudan.

**Methods:**

This cross-sectional study was conducted during April-July 2016 at different hospitals in Khartoum State. Mid-stream urine samples were obtained from 150 hospitalized pregnant women and cultured on CLED (Cystine Lactose Electrolyte Deficient) agar. Microorganisms were identified using standard microbiological procedures. Isolated Gram-negative bacteria were tested for antibiotic susceptibility and ESBL screening using modified Kirby- Bauer method and Double Disc Synergy Test (DDST) respectively.

**Results:**

Urine culture revealed positive results in 33/150 (22%) and the most prevalent isolates were Gram negative bacteria (18/33, 54.5%). Among gram-negative bacteria, isolates of *E. coli* were the most prevalent accounting 66.6% (12/18) followed by *K. pneumoniae* (4/18, 22.2%) and *K. oxytoca* (2/18, 11.1%). ESBL was detected in 8/18 (44.4%) of the Gram-negative isolates. Of note, imipenem was the most susceptible antibiotic for ESBL-producer and non-ESBL producer Gram negative isolates, accounting 100% susceptibility for both bacterial groups. Overall susceptibility rates were also high for ciprofloxacin (13/18, 72.2%). In other hand, co-trimoxazole and amoxicillin showed high resistance pattern for ESBL-producer and non-ESBL producer isolates; 27.8%, 44.4% and 38.9%, 38.9% susceptibility rates of co-trimoxazole and amoxicillin for ESBL-producer and non-ESBL producer isolates, respectively.

**Conclusions:**

Imipenem remains the most powerful option for ESBL- and non-ESBL bacteria causing UTIs in pregnant women. However, due to tremendous increase of antibiotic-resistant, antibiotic-susceptibility testing is recommended as a routine investigation for admitted pregnant women.

## INTRODUCTION

Urinary tract infection (UTI) represents the most common bacterial infection in pregnancy and the third common cause of human infections after respiratory and intestinal infections. Depending on the infected site and other factors predispose individuals to UTI, the infection can be life threatening and associated with serious complications.^1^

Urinary tract infection can be associated with defined symptoms ‘symptomatic’ or without symptoms ‘asymptomatic’. Asymptomatic bacteriuria is defined as growth of bacteria in the urinary tract of individuals without signs or symptoms associated with urinary or genital organs. It considered a frequent health problem among pregnant women, occurring within 2–10% of all pregnancies.^2^-^6^ If not properly treated, asymptomatic bacteriuria can lead to acute pyelonephritis in 30% of pregnant women^7^ and increase the risk for severe complication such as early delivery, hypertension, pre-eclampsia, low birth weight and postpartum endometritis.^8^,^9^

*E*. *coli* accounts for most cases of symptomatic and asymptomatic bacteriuria in women, representing 70–90% of the cases.^10^ This organism is usually originating from gut micro flora, colonizes the vagina and disseminates and causing infection in urinary organs. *E. coli causing UTI*, in particular, have several virulence factors discriminating them from gut flora and playing essential role in the pathogenesis of the disease. *E. coli* virulence factors include the surface component ‘adhesin’ and a variety of iron sequestration systems.^11^ Pathogens of urinary tract include other Gram-negative bacilli such as *Klebsiella spp. and Pseudomonas spp*, Gram Positive cocci such as *S. saprophyticus* and *Enterococcus spp.^12^,^13^*

Gram negative bacteria often acquire genes coding for antibiotic resistance, in particular under antibiotic selection pressure. β-lactamase enzymes are the most frequent and play a key role, conferring resistance of bacteria to the β-Lactam antibiotic group, such as penicillins and cephalosporins.^14^,^15^ Antibiotic resistances among Gram negative bacteria is on increase due to the spread of strains producing extended-spectrum β-lactamase enzymes (ESBLs). ESBL’s coding genes are carried on bacterial chromosomes that can be inherited or acquired through plasmid with the possibility to move between bacterial populations. They are able to inactivate third-generation cephalosporins and aztreonam, but can be inhibited by clavulanic acid.^16^

In Sudan, there are few studies focused on antibiotics resistance pattern by Gram negative bacilli and identification of risk factors for ESBL infection among pregnant women remain unclear.^17^ Urine for culture and susceptibility to antimicrobials is not routinely done for pregnant women suffering from UTI. In most cases, antibiotics are prescribed blindly without sensitivity testing for the causative agents. The present study helps physician prescribing appropriate antibiotic therapy and reduces potential subsequent complications of UTI during pregnancy.

## METHODS

Across-sectional study was conducted during April-July 2016 at different hospitals in Omdurman, Khartoum State, Sudan. Three hospitals were randomly selected for the study; Omdurman Maternity Hospital, Omdurman New Hospital and Omdurman Military Hospital (Obstetrics and Gynecology department). All pregnant women who admitted to antenatal wards were invited to participate in this study. One hundred and fifty (n=150) pregnant women agreed to participate in this study and signed a consent Form. A questionnaire was used for collection of primary data include participant age, pregnancy trimester, signs and symptoms of UTI, contraceptive use, recurrent UTI episodes, previous antibiotic intake and medical history of diabetes mellitus, hypertension and anemia. The population was divided into two groups, symptomatic and asymptomatic, based on the presence of UTI signs and symptoms.

### Isolation and identification

Mid-stream urine samples were collected and transported on ice to the Research Laboratory of Sudan University of Science and Technology. Under aseptic conditions, all urine samples were cultured on Cysteine Lactose Electrolytes Deficient media, CLED (HiMedia, India) and incubated aerobically for 18-24 hrs at 37°C. Primary identification of isolates was done by colonial features and Gram stain. Suspected organisms were identified by conventional biochemical tests described earlier.^18^ Identification tests included citrate utilization test, urease test, indole test, catalase test, DNAse test, Mannitol fermentation test, Esculin hydrolysis test and inoculation of KIA media to test for sugar fermentation and H_2_S.

### Ethical consideration

The study was technically and ethically approved by the Research Board at Sudan University of Science and Technology and the Ministry of Health in Sudan (No. 2015/3/2 and 2015/3/22). All Participants were signed written informed consents before enrolling in the study.

### Antimicrobial susceptibility test

A modified Kirby- Bauer susceptibility testing method^19^,^20^ was used to assess the sensitivity and resistance patterns of Gram negative uropathogenic isolates. A set of antibiotics discs were applied include imipenem 10µg, ciprofloxacin 30µg, co-trimoxazole 30µg, amoxicillin 30µg, cefuroxime 30µg, ceftazidime 30µg, cefotaxime 30µg and ceftriaxone 30µg. All antibiotic discs were purchased from HiMedia, India. Culture plates were incubated aerobically for overnight at 37°C. All Gram-negative bacilli isolates which showed a diameter of inhibition zone less than 17 mm for ceftazidime, less than 19 mm for ceftriaxone and less than 22 mm for cefotaxime were selected for testing ESBL enzyme production using Double Disc Synergy Test (DDST) as previously reported.

### Double Disc Synergy Test (DDST)

A suspension of tested isolate was compared with McFarland standard (0.5%) and plated on Mueller Hinton agar. Amoxicillin/clavulanic acid (20/10µg) disc was placed in the center of the plate and ceftazidime (30µg) and cefotaxime (30µg) discs were placed 15 mm apart center to center to amoxicillin/clavulanic acid and incubated aerobically for 18-24 hrs at 37°C.^21^ Any increase in the inhibition zone towards the disc of amoxicillin/clavulanic acid was considered as positive result for ESBL enzyme production.

### Statistical Analysis

SPSS version 16 was used for analysis of the results. Pearson Chi-square test and odds ratio (OR) were used to assess risk factors associated with ESBL infection. OR was presented as ratio number at 95% confidence interval (CI) and was assessed as follow: OR= 1, no association between variables and infection; OR>1, association with higher odds of infection; <1, association with lower odds of infection.^22^

## RESULTS

Characteristics and clinical features of the participants were studies (Table-I). Age group 26-36 years represented 56% (84/150) of participants and pregnant women in third trimester accounted for most of the study population (142/150, 94.7%). Medical conditions such as contraceptive use (39/150, 26%), UTI recurrence (82/150, 54.7%) and previous antibiotics intake (78/150, 52%) showed high distributions among the participants. Diabetes mellitus, hypertension and anemia were observed in 9.3% (14/150), 10.7% (16/150) and 14% (21/150) of the pregnant women, respectively.

**Table I:** Characteristics of patients screened for ESBL infection.

Characteristic	Number (%)
*Age group*	
15-25years	41 (27.3)
26-36 years	84 (56)
37-47 years	25 (16.7)
*Pregnancy trimester*	
Second trimester	8 (5.3)
Third trimester	142 (94.7)
*Medical conditions*	
Contraceptive use	39 (26)
Recurrence UTI	82 (54.7)
Previous Antibiotic intake	78 (52)
Diabetic mellitus	14 (9.3)
Hypertension	16 (10.7)
Anemia	21 (14)

ESBL: Extended Spectrum Beta Lactamases

Among the 150 studied pregnant women, positive urine cultures were reported in 33 (22%). Majority of pregnant women with UTIs were asymptomatic; 66.7% and 33.3% for asymptomatic and symptomatic patients, respectively. Most prevalent isolates were Gram negative bacteria (18/33, 54.5%). *E. coli* was the most prevalent isolates accounting 66.6% (12/18) followed by *K. pneumoniae* (4/18, 22.2%) and *K. oxytoca* (2/18, 11.1%).

Resistance patterns to third generation cephalosporins were assessed in 18 Gram negative bacilli isolates, including *E. coli* (n=12), *K. pneumoniae* (n=4) and *K. oxytoca* (n=2). Cefuroxime was the highest resistance (100%) followed by moderate resistance for ceftazidime, cefotaxime and ceftriaxone; accounting 55.6%, 61.1% and 61.1%, respectively. Ten isolates of Gram negative with resistance to ceftazidime, cefotaxime and ceftriaxone were selected to detect ESBL enzyme by using DDST. Phenotypic method of ESBL in Gram negative uropathogenic bacteria detected 8 (44.4%) potential positive isolates and *E. coli* was the most prevalent ESBL isolates accounting 75% (6/8).

Antimicrobial susceptibility of ESBL-producer and non-ESBL producer isolates revealed that imipenem was the most susceptible antibiotic for both ESBL-producer and non-ESBL producer Gram negative isolates, accounting 100% susceptibility for both groups (Table-II). Overall susceptibility rates were also high for ciprofloxacin (72.2%). In other hand, co-trimoxazole and amoxicillin showed high resistance pattern for both ESBL-producer and non-ESBL producer isolates; 27.8%, 44.4% and 38.9%, 38.9% susceptibility rates of co-trimoxazole and amoxicillin for ESBL-producer and non-ESBL producer Gram negative bacteria, respectively.

**Table II:** Antibiotics susceptibility patterns of ESBL-producer and non-ESBL producer Gram negative isolates (n=18).

Antibiotics	Susceptibility patterns				

	Sensitive	Intermediate	Resistant		

			ESBL-producer	Non-ESBL producer	Total
IPM	18 (100%)	0 (0%)	0 (0%)	0 (0%)	0 (0%)
CIP	13 (72.2%)	1 (5.6%)	2 (11.1%)	2 (11.1%)	4 (22.2%)
STX	6 (33.3%)	0 (0%)	5 (27.8%)	7 (38.9%)	12 (66.7%)
AMC	2 (11.1%)	1 (5.6%)	8 (44.4%)	7 (38.9%)	15 (83.3%)

ESBL: Extended Spectrum β- Lactamase producer, IPM: Imipenem, CIP: Ciprofloxacin, STX: Co-trimoxazole, AMC: Amoxicillin. Values are numbers and percentages of Gram negative isolates according to their susceptibility patterns to different antibiotics.

The risk for acquiring ESBL-producing bacteria was found to be higher in pregnant women in second trimester (OR=2.755; 95% CI =0.297-25.591) and contraceptive users (OR =1.1767; 95% CI= 0.402-7.764), while it was found to be lower among pregnant women with recurrent UTI (OR = 0.821; 95% CI=0.197-3.411) and antibiotic users (OR= 0.919; 95% CI=0.221-3.819), with almost no difference among diabetic, hypertensive and anemic patients compared to their counterparts (Table-III).

**Table III:** Risk estimation for acquiring ESBL infection.

Variable	OR (95% CI)
*Pregnancy trimester*	
Second trimester	2.755 (0.297 -25.59)
Third trimester	1
*Contraceptive use*	
Yes	1.767(0.402 to 7.764)
No	1
*Recurrence UTI*	
Yes	0.821(0.197 to 3.411)
No	1
*Previous Antibiotic intake*	
Yes	0.919 (0.221 to 3.819)
No	1
*Diabetic mellitus*	
Yes	1.062 (1.019 to 1.108)
No	1
*Hypertension*	
Yes	1.063 (1.019 to 1.110)
No	1
*Anemia*	
Yes	1.066 (1.020 to 1.114)
No	1

## DISCUSSION

Symptomatic or asymptomatic urinary tract infections are the most common infection among pregnant women and can lead to serious complications, if not treated.^4^ In our study, prevalence of symptomatic UTI among pregnant women was 33.3%. This percentage was similar to previous reports from similar regions. Symptomatic UTI was reported in 28% and 31.59% in Sudan and Tanzania, respectively. On other hand, prevalence of UTI in asymptomatic infection was 66.7%, which was also in agree with publications reported earlier; 71.9% and 68.4% prevalence of asymptomatic UTI.^23^,^24^ Indeed, pregnant women have increased risk for asymptomatic UTI that can progress to pyelonephritis and other serious adverse outcomes.^7^ Such complications can be avoided through implementing relevant investigation tests for UTI among pregnant women.

Nowadays, concerns about antibiotic resistance have been increased particularly among Gram negative pathogens producing the ESBL enzymes. One of the most important issue facing hospitals and health care settings is the increased number of ESBL producing bacteria, most of these bacteria have been isolated from critical patients.^16^,^17^ Here, we reported ESBL producers in 44.4% of Gram-negative isolates. Globally, the frequency of ESBL-producer bacteria varies in the different countries. It has been shown that prevalence of ESBL among *K. pneumoniae* was high in Latin America (44%) as compared to Asia, Europe and North America (7.5-22.4%).^25^,^26^ Other studies done in India^27^ and Uganda^28^ showed ESBL producers in 57.2% and 62%, respectively. These studies included wards patients that can explain the aspects of similarity. In African countries, the situation about ESBL-producer bacteria is greatly poor.^23^ This vague situation calls the urgent need for implementing programs targeting detection of drug resistance bacteria and investigating ESBL production among Gram negative bacteria.

We also showed that *E. coli* was the most prevalent ESBL producing isolates (75%). This finding was different from previous studies where *Klebsiella spp* was found to be the most prevalent bacteria.^23^ A study done in Taiwan reported ESBLs producer *E. coli* as 1.5-16.7%.^29^ *E.coli* is the most common bacteria causing UTI among pregnant women, thus female gender and pregnancy in this study played a role in the increased percentage of *E. coli* producing ESBL in comparison with other studies. ESBLs producing *K. pneumoniae* represent 25% in present study. This result was less than other studies showed 67.04% (38) and 72.7% (28), whereas it agrees with Yu and his colleagues, reporting 8.5-29.8%.^29^ This low percentage of ESBLs producing *K.pneumoniae* in our study may refer to low number of *K. pneumoniae* isolates due to the small sample size.

It is not surprising that all uropathogens isolated from pregnant women including ESBL-producers and non-ESBL producers were 100% susceptible to imipenem. Earlier reports have described imipenem as the drug of choice for complicated bacterial infections such as UTI.^30^,^31^ This antibiotic has a broad-spectrum action against wide group of pathogens such as Gram positive and Gram negative bacteria. Because of its safety and effectiveness for treatment of bacterial infection, we recommend continue use of imipenem as a therapy for severe infections among pregnant women.

Many predisposing factors contribute in UTI development such as age, female gender, sexual activity, pregnancy, contraception, instrumentation, urinary tract obstruction, neurologic dysfunction, previous antibiotics use and other factors.^32^ Here, we reported that pregnant women in second trimester and former contraceptive users are in high risk for ESBL infection. Indeed, use of contraceptives increase UTI risks and the potential for acquiring ESBL-producing bacterial will also increase. The association been previous contraceptive use and UTIs has been extensively studied. 33 We did not observe any relationship between diabetes mellitus, hypertension or anemia to ESBL infection acquisition.

## CONCLUSION

Our finding revealed high prevalence of asymptomatic bacteriuria among hospitalized pregnant women in Omdurman hospital. ESBLs production was found to be high among Gram negative urinary isolates. Imipenem remain the most effective treatment for ESBL-producer and non-ESBL producer bacteria causing UTIs in pregnant women. Detection of ESBL among Gram negative bacteria would better guide antibiotic therapy and achieve better treatment outcome. The main limitation of the study is the small population size. A bigger population would be required to validate risks for infection with ESBL. Molecular methods would have also validated our results.
